# Author Correction: Systematic assessment of ISWI subunits shows that NURF creates local accessibility for CTCF

**DOI:** 10.1038/s41588-024-01985-3

**Published:** 2024-11-01

**Authors:** Mario Iurlaro, Francesca Masoni, Ilya M. Flyamer, Christiane Wirbelauer, Murat Iskar, Lukas Burger, Luca Giorgetti, Dirk Schübeler

**Affiliations:** 1 Friedrich Miescher Institute for Biomedical Research, Basel, Switzerland; 2 Faculty of Science, University of Basel, Basel, Switzerland; 3 Swiss Institute of Bioinformatics, Basel, Switzerland; 4 Present Address: Disease Area Oncology, Novartis Biomedical Research, Basel, Switzerland

**Keywords:** Epigenetics, Gene regulation, Epigenomics

Correction to: *Nature Genetics* 10.1038/s41588-024-01767-x, published online 30 May 2024.

In the version of the article initially published, there was a mistake in the amplicon annotation used for footprinting which led to the wrong pairing of some SMF amplicons to the corresponding genomic locus. Fig. 3b and Supplementary Table 8 have therefore been corrected, and the original and corrected Fig. 3b can be seen in Fig. 1. Results and conclusions are not affected. In the “ATAC-seq data analysis” section of the Methods, “*P* value > 100” has replaced “*P* value < 0.001” in the sentence now reading “For visualization, motifs that had a log_2_ fold enrichment of >1.5 and −log_10_-adjusted *P* value > 100”. In the “CUT&RUN data analysis” section of the Methods, a citation to “Extended Data Fig. 6e,g” has been replaced with “Extended Data Fig. 5e,g”. Additionally, in seven instances in the Methods, when a cell number was listed with an exponential annotation it was wrongly presented during processing with a negative exponent, all of which have now been removed. These corrections have been made to the HTML and PDF versions of the article.

**Fig. 1:**
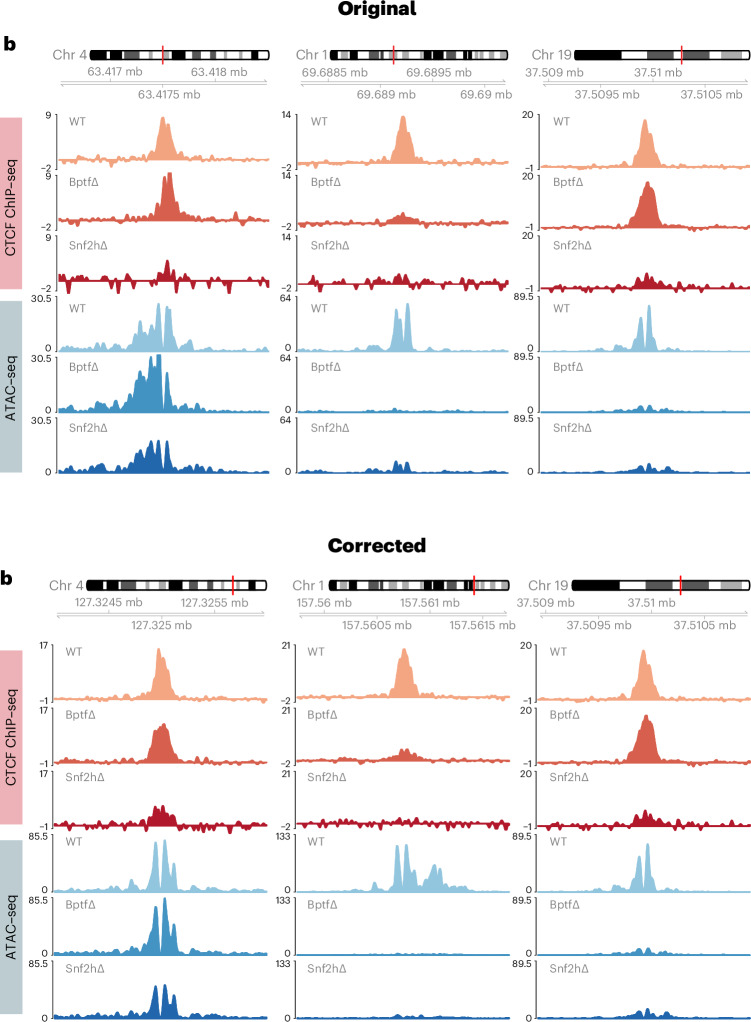
Original and corrected Fig. 3b.

