# Supplementary material for: Systematic assessment of ISWI subunits shows that NURF creates local accessibility for CTCF
Source: Nat Genet. 2024 May 30;56(6):1203–12. doi: 10.1038/s41588-024-01767-x (PMC11176080; doi:10.1038/s41588-024-01767-x)
Supplement: Supplementary file 1 — Supplementary Figs. 1 and 2. [file 41588_2024_1767_MOESM1_ESM.pdf]

nature genetics

Supplementary information

https://doi.org/10.1038/s41588-024-01767-x

Systematic assessment of ISWI subunits shows that NURF creates local accessibility for CTCF

In the format provided by the authors and unedited

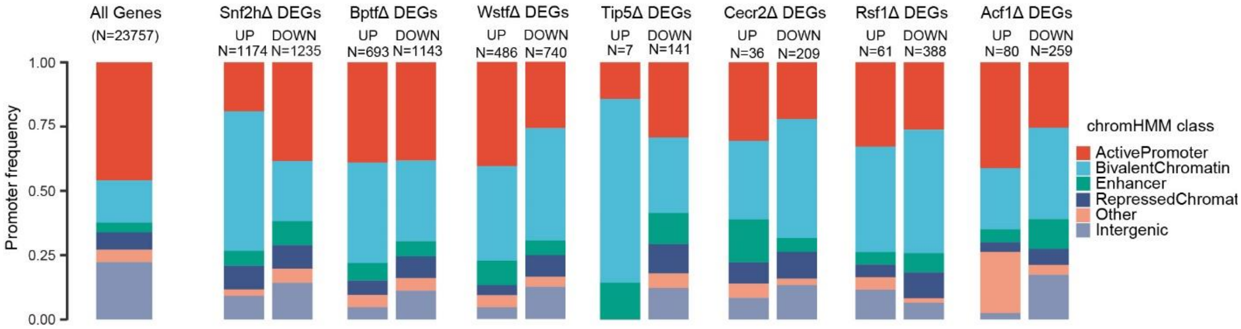

Supplementary Figure 1. Chromatin states of DEGs promoters

Distribution of chromatin states at promoters of all genes and DEGs for each deletion line, as labeled by chromHMM and split by upregulated and downregulated genes.

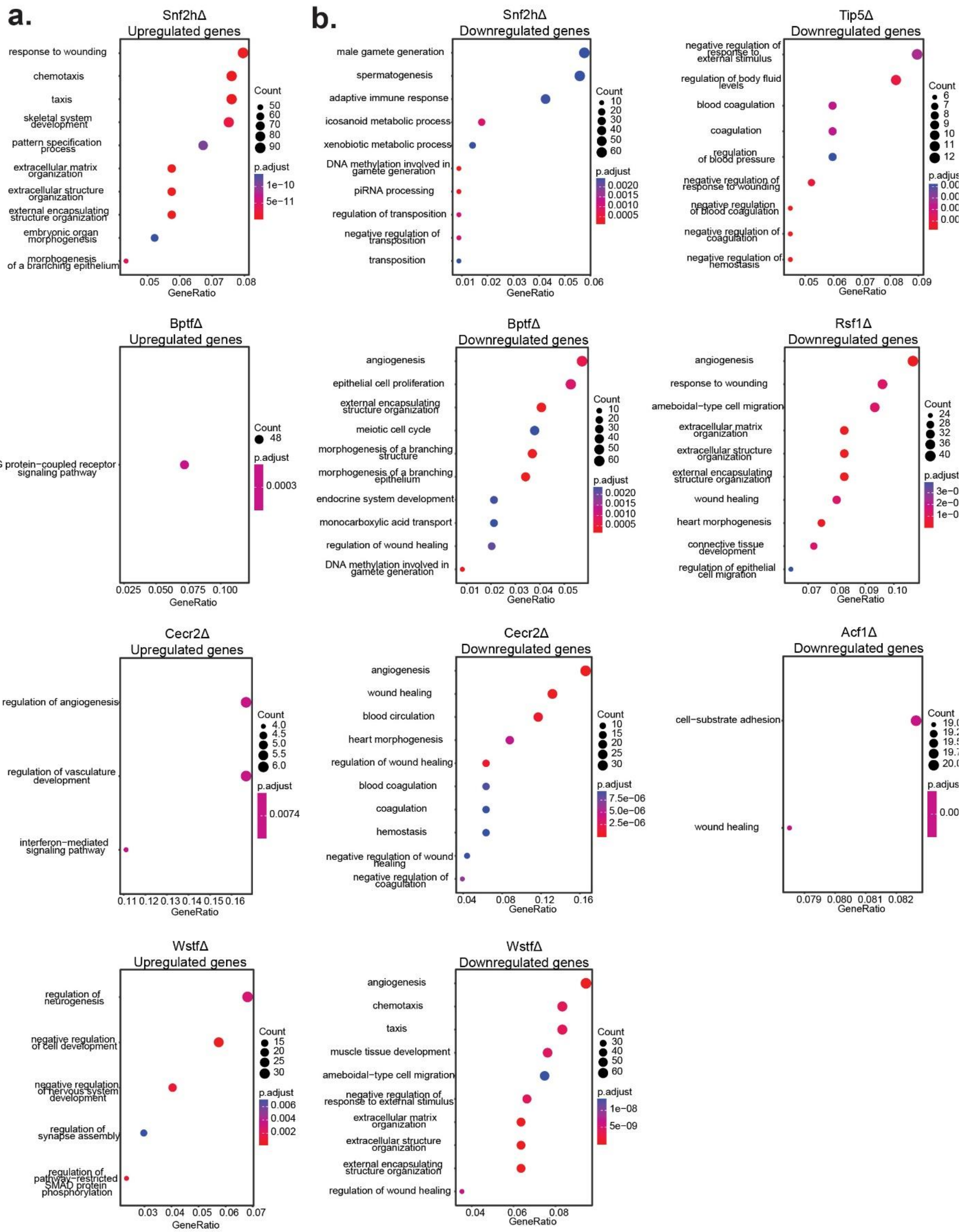
a.
b.
Snf2hΔ
Upregulated genes
response to wounding
chemotaxis
taxis
skeletal system development
pattern specification process
extracellular matrix organization
extracellular structure organization
external encapsulating structure organization
embryonic organ morphogenesis
morphogenesis of a branching epithelium
Count
p.adjust
GeneRatio
Snf2hΔ
Downregulated genes
male gamete generation
spermatogenesis
adaptive immune response
icosanoid metabolic process
xenobiotic metabolic process
DNA methylation involved in gamete generation
piRNA processing
regulation of transposition
negative regulation of transposition
transposition
Tip5Δ
Downregulated genes
negative regulation of response to external stimulus
regulation of body fluid levels
blood coagulation
coagulation
regulation of blood pressure
negative regulation of response to wounding
negative regulation of blood coagulation
negative regulation of coagulation
negative regulation of hemostasis
BptfΔ
Upregulated genes
G protein−coupled receptor signaling pathway
BptfΔ
Downregulated genes
angiogenesis
epithelial cell proliferation
external encapsulating structure organization
meiotic cell cycle
morphogenesis of a branching structure
morphogenesis of a branching epithelium
endocrine system development
monocarboxylic acid transport
regulation of wound healing
DNA methylation involved in gamete generation
Rsf1Δ
Downregulated genes
angiogenesis
response to wounding
ameboidal−type cell migration
extracellular matrix organization
extracellular structure organization
external encapsulating structure organization
wound healing
heart morphogenesis
connective tissue development
regulation of epithelial cell migration
Cecr2Δ
Upregulated genes
regulation of angiogenesis
regulation of vasculature development
interferon−mediated signaling pathway
Cecr2Δ
Downregulated genes
angiogenesis
wound healing
blood circulation
heart morphogenesis
regulation of wound healing
blood coagulation
coagulation
hemostasis
negative regulation of wound healing
negative regulation of coagulation
Acf1Δ
Downregulated genes
cell−substrate adhesion
wound healing
WstfΔ
Upregulated genes
regulation of neurogenesis
negative regulation of cell development
negative regulation of nervous system development
regulation of synapse assembly
regulation of pathway−restricted SMAD protein phosphorylation
WstfΔ
Downregulated genes
angiogenesis
chemotaxis
taxis
muscle tissue development
ameboidal−type cell migration
negative regulation of response to external stimulus
extracellular matrix organization
extracellular structure organization
external encapsulating structure organization
regulation of wound healing

Supplementary Figure 2. Gene Ontology analysis on differentially expressed genes in ISWI subunit deletions.

a. Gene Ontology (see Methods) terms enriched among the set of upregulated genes of ISWI deletion lines. Adjusted p-value calculated through a one-sided Fisher's exact test with Benjamini-Hochberg multiple testing correction. **b**. Gene Ontology terms enriched among the set of downregulated genes of ISWI deletion lines. Adjusted p-value was calculated as in **a**.
