# Supplementary material for: Systematic assessment of ISWI subunits shows that NURF creates local accessibility for CTCF
Source: Nat Genet. 2024 May 30;56(6):1203–12. doi: 10.1038/s41588-024-01767-x (PMC11176080; doi:10.1038/s41588-024-01767-x)
Supplement: Supplementary file 2 — Reporting Summary [file 41588_2024_1767_MOESM2_ESM.pdf]

nature portfolio

Corresponding author(s): Dirk Schübeler

Last updated by author(s): Mar 18, 2024

Reporting Summary

Nature Portfolio wishes to improve the reproducibility of the work that we publish. This form provides structure for consistency and transparency in reporting. For further information on Nature Portfolio policies, see our Editorial Policies and the Editorial Policy Checklist.

Statistics

For all statistical analyses, confirm that the following items are present in the figure legend, table legend, main text, or Methods section.

n/a	Confirmed	
☐	☒	The exact sample size (*n*) for each experimental group/condition, given as a discrete number and unit of measurement
☐	☒	A statement on whether measurements were taken from distinct samples or whether the same sample was measured repeatedly
☐	☒	The statistical test(s) used AND whether they are one- or two-sided<br>*Only common tests should be described solely by name; describe more complex techniques in the Methods section.*
☐	☒	A description of all covariates tested
☐	☒	A description of any assumptions or corrections, such as tests of normality and adjustment for multiple comparisons
☐	☒	A full description of the statistical parameters including central tendency (e.g. means) or other basic estimates (e.g. regression coefficient) AND variation (e.g. standard deviation) or associated estimates of uncertainty (e.g. confidence intervals)
☐	☒	For null hypothesis testing, the test statistic (e.g. *F*, *t*, *r*) with confidence intervals, effect sizes, degrees of freedom and *P* value noted<br>*Give P values as exact values whenever suitable.*
☒	☐	For Bayesian analysis, information on the choice of priors and Markov chain Monte Carlo settings
☐	☒	For hierarchical and complex designs, identification of the appropriate level for tests and full reporting of outcomes
☐	☒	Estimates of effect sizes (e.g. Cohen's *d*, Pearson's *r*), indicating how they were calculated

Our web collection on statistics for biologists contains articles on many of the points above.

Software and code

Policy information about availability of computer code

Data collection	Software used for data collection: For ChIPseq, Illumina RTA 1.18.64 and bcl2fastq2 v2.17 was used for basecalling and demultiplexing. For RNAseq, Illumina RTA 1.18.64 and bcl2fastq2 v2.17 was used for basecalling and demultiplexing samples generated by Illumina HiSeq sequencing. Illumina RTA 3.4.4 and bcl2fastq2 v2.20 was used for basecalling and demultiplexing samples generated by Illumina NovaSeq sequencing. For ATACseq, Illumina RTA 2.4.11 and bcl2fastq2 v2.17 was used for basecalling and demultiplexing samples generated by Illumina NextSeq. Illumina RTA 3.4.4 and bcl2fastq2 v2.20 was used for basecalling and demultiplexing samples generated by Illumina NovaSeq. For MNaseseq, Illumina RTA 2.4.11 and bcl2fastq2 v2.17 was used for basecalling and demultiplexing samples generated by Illumina NextSeq. Illumina RTA 3.4.4 and bcl2fastq2 v2.20 was used for basecalling and demultiplexing samples generated by Illumina NovaSeq sequencing. For CUT&RUN, Illumina RTA 2.4.11 and bcl2fastq2 v2.17 was used for basecalling and demultiplexing. Protein identification and relative quantification was performed with MaxQuant v.1.5.3.8.
Data analysis	When required adaptor removal for genomic experiments was performed using cutadapt_2.5. Data analysis for genomic experiments was performed using R 4.3.0 and R/Bioconductor packages: BSgenome.Mmusculus.UCSC.mm10_1.4.0, TxDb.Mmusculus.UCSC.mm10.knownGene 3.10.0, QuasR_1.40.1, RBowtie_1.40.0, ComplexHeatmap_2.12.0., clusterProfiler 4.8.1, edgeR 3.40.2, MACS2_2.2.7.1, swissknife 0.40, ggseqlogo_1.0, Keras_2.2.5.0,TensorFlow_ 2.0.0, JASPAR2022 0.99.7, monaLisa 1.6.0. For proteomics experiments protein identification and relative quantification was performed using MaxQuant_1.5.3.8, limma_3.56.2 was used for statistics. For Hi-C experiments, data analysis was peformed using using Python 3.9.7 and packages: BWA 0.7.17, cooler 0.9.0, cooltools 0.6.1, mustache 1.3.2, coolpup.py 1.1.0.

For manuscripts utilizing custom algorithms or software that are central to the research but not yet described in published literature, software must be made available to editors and reviewers. We strongly encourage code deposition in a community repository (e.g. GitHub). See the Nature Portfolio guidelines for submitting code & software for further information.

Data

Policy information about availability of data

All manuscripts must include a data availability statement. This statement should provide the following information, where applicable:

- Accession codes, unique identifiers, or web links for publicly available datasets
- A description of any restrictions on data availability
- For clinical datasets or third party data, please ensure that the statement adheres to our policy

Next-generation sequencing data generated in this study are available at Gene Expression Omnibus (GEO, https://www.ncbi.nlm.nih.gov/geo/) with accession no. GSE234295 and GSE250229. The following public datasets were obtained from GEO: histone marks ChIP–seq: H3K27me3: (GSE30203, samples GSM747539 to GSM747541) (ref. 75), H3K4me1: (GSE30203, sample GSM747542) (ref.75), H3K27ac: (GSE67867, samples GSM1891651 and GSM1891652) (ref.76), H3K36me3: (GSE33252, samples GSM801982 and GSM801983) (ref. 77); Hi-C CTCF-AID-UNT and CTCF-AUX-48h (GSE98671, samples GSM2644945 to GSM2644948) (ref.30); ChIP-seq CTCF-AID-UNT and CTCF-AUX-48h (GSE98671, samples GSM2609185 and GSM2609186) (ref. 30); SNF2H knockout: (GSE112136 Hi-C samples GSM3331341 to GSM3331344, MNase samples GSM3058339 to GSM3058342, RNA-seq samples GSM3058347 to GSM3058359, ChIP-seq samples GSM3058327 and GSM3058328) (ref.14). The UCSC annotation of known genes for mm10 was obtained through the Bioconductor annotation package TxDb.Mmusculus.UCSC.mm10.knownGene (10.18129/B9.bioc.TxDb.Mmusculus.UCSC.mm10.knownGene). The Jaspar2022 (ref.56) motif database used in this study can be accessed online (https://jaspar2022.genereg.net/).
The mass spectrometry proteomics data generated in this study have been deposited to the ProteomeXchange Consortium via the PRIDE78 partner repository with the dataset identifier PXD042945.
Source data are provided with this paper.

Research involving human participants, their data, or biological material

Policy information about studies with human participants or human data. See also policy information about sex, gender (identity/presentation), and sexual orientation and race, ethnicity and racism.

Reporting on sex and gender	n/a
Reporting on race, ethnicity, or other socially relevant groupings	n/a
Population characteristics	n/a
Recruitment	n/a
Ethics oversight	n/a

Note that full information on the approval of the study protocol must also be provided in the manuscript.

Field-specific reporting

Please select the one below that is the best fit for your research. If you are not sure, read the appropriate sections before making your selection.

☒ Life sciences ☐ Behavioural & social sciences ☐ Ecological, evolutionary & environmental sciences

For a reference copy of the document with all sections, see nature.com/documents/nr-reporting-summary-flat.pdf

Life sciences study design

All studies must disclose on these points even when the disclosure is negative.

Sample size	No statistical method was used to pre-determine sample size. All experiment were performed in at least 2 independent replicates in line with accepted practice in the genomics field.
Data exclusions	No data was excluded.
Replication	All experiments were performed in 2-4 biological replicates as indicated in the manuscript. All attempts at replication were successful.
Randomization	The study conditions did not require randomization as this study design is not affected by the order of recording for experimental data.
Blinding	The study conditions did not require blinding as this study design is not affected by the observer carrying out the experiments.

Reporting for specific materials, systems and methods

We require information from authors about some types of materials, experimental systems and methods used in many studies. Here, indicate whether each material, system or method listed is relevant to your study. If you are not sure if a list item applies to your research, read the appropriate section before selecting a response.

Materials & experimental systems

n/a	Involved in the study
☐	☒ Antibodies
☐	☒ Eukaryotic cell lines
☒	☐ Palaeontology and archaeology
☒	☐ Animals and other organisms
☒	☐ Clinical data
☒	☐ Dual use research of concern
☒	☐ Plants

Methods

n/a	Involved in the study
☐	☒ ChIP-seq
☒	☐ Flow cytometry
☒	☐ MRI-based neuroimaging

Antibodies

Antibodies used

Snf2h-ab72499-Abcam, Snf2h-ab3749-Abcam, Snf2l-D4Q7V-CellSignaling, CTCF-C-20X-Santa Cruz, CTCF-ab128873-Abcam, Rad21-ab992-Abcam, Rad21-154769-Abcam, Wapl-16370-1-AP-Proteintech, IgG-M7023_Sigma Aldrich, Acf1-A301-318A-Bethyl Laboratories, Cecr2-LSC-496852-LSBio, Wstf-ab51256-Abcam,
Tip5-C15310090-Diagenode, Bptf-ABE24-Millipore, Rsf1-ab109002-Abcam, LaminB-ab16048-Abcam.

Validation

all antibodies were validated by the manufacturer.
ab72499 https://www.abcam.com/products/primary-antibodies/snf2h-antibody-ab72499.html
D4Q7V https://www.cellsignal.com/products/primary-antibodies/smarca1-d4q7v-rabbit-mab/12483
ab3749 https://www.abcam.com/products/primary-antibodies/snf2h-antibody-chip-grade-ab3749.html
C-20X https://www.scbt.com/p/ctcf-antibody-c-20
ab128873 https://www.abcam.com/products/primary-antibodies/ctcf-antibody-epr7314b-chip-grade-ab128873.html
ab992 https://www.abcam.com/products/primary-antibodies/rad21-antibody-ab992.html
ab154769 https://www.abcam.com/products/primary-antibodies/rad21-antibody-ab154769.html
16370-1-AP https://www.thermofisher.com/antibody/product/WAPL-WAPAL-Antibody-Polyclonal/16370-1-AP
M7023 https://www.sigmaaldrich.com/CH/en/product/sigma/m7023
A301-318A https://www.thermofisher.com/antibody/product/ACF1-BAZ1A-Antibody-Polyclonal/A301-318A
LSC-496852 https://www.lsbio.com/antibodies/cecr2-antibody-wb-western-ls-c496852/510169
ab51256 https://www.abcam.com/products/primary-antibodies/wstf-antibody-ep1704y-ab51256.html
C15310090 https://www.diagenode.com/en/p/tip-5-polyclonal-antibody-classic-100-ul
ABE24 https://www.merckmillipore.com/CH/de/product/Anti-BPTF-Antibody,MM_NF-ABE24
ab109002 https://www.abcam.com/products/primary-antibodies/rsf1-antibody-epr37492-ab109002.html
ab16048 https://www.abcam.com/products/primary-antibodies/lamin-b1-antibody-nuclear-envelope-marker-ab16048.html

Eukaryotic cell lines

Policy information about cell lines and Sex and Gender in Research

Cell line source(s)

Mouse ES cell line of 129S6/SvEvTac background was originally obtained from Miriam Bibel and is described in PMID 17546008. Snf2hΔ line was generated in this background and is described in PMID 30996347. The new lines generated in this study namely Acf1Δ, Rsf1Δ, Tip5Δ, Cecr2Δ, WstfΔ,BptfΔ and Snf2lΔ were generated in the same background and are available upon request.

Authentication

Genotype of cell lines was tested at the level of DNA sequence and protein.

Mycoplasma contamination

Cell lines tested negative for Mycoplasm.

Commonly misidentified lines
(See ICLAC register)

No commonly misidentified cell lines were used.

Plants

Seed stocks

/

Novel plant genotypes

/

Authentication

/

ChIP-seq

Data deposition

☒ Confirm that both raw and final processed data have been deposited in a public database such as GEO.

☐ Confirm that you have deposited or provided access to graph files (e.g. BED files) for the called peaks.

Data access links<br>*May remain private before publication.*	https://www.ncbi.nlm.nih.gov/geo/query/acc.cgi?acc=GSE234295 (SuperSeries - all data)<br>https://www.ncbi.nlm.nih.gov/geo/query/acc.cgi?acc=GSE234273 (ChIP-seq data)<br><br>https://www.ncbi.nlm.nih.gov/geo/query/acc.cgi?acc=GSE250229 (SuperSeries all data)<br>https://www.ncbi.nlm.nih.gov/geo/query/acc.cgi?acc=GSE250226 (ChIP-seq)
Files in database submission	For each ChIP-seq sample, the GEO entry contains two files: the rawdata (fastq format) and a file with alignment density per 100 bp in the mouse mm10 genome (wig file).<br><br>fastq files:<br>CTCF_input_BPTFko_rep1_1.fastq.gz<br>CTCF_input_BPTFko_rep2_1.fastq.gz<br>CTCF_input_BPTFko_rep3_1.fastq.gz<br>CTCF_input_BPTFko_rep3_2.fastq.gz<br>CTCF_input_BPTFko_rep4_1.fastq.gz<br>CTCF_input_BPTFko_rep4_2.fastq.gz<br>CTCF_input_wt_rep1_1.fastq.gz<br>CTCF_input_wt_rep2_1.fastq.gz<br>CTCF_input_wt_rep3_1.fastq.gz<br>CTCF_input_wt_rep3_2.fastq.gz<br>CTCF_input_wt_rep4_1.fastq.gz<br>CTCF_input_wt_rep4_2.fastq.gz<br>CTCF_IP_BPTFko_rep1_1.fastq.gz<br>CTCF_IP_BPTFko_rep2_1.fastq.gz<br>CTCF_IP_BPTFko_rep3_1.fastq.gz<br>CTCF_IP_BPTFko_rep3_2.fastq.gz<br>CTCF_IP_BPTFko_rep4_1.fastq.gz<br>CTCF_IP_BPTFko_rep4_2.fastq.gz<br>CTCF_IP_wt_rep1_1.fastq.gz<br>CTCF_IP_wt_rep2_1.fastq.gz<br>CTCF_IP_wt_rep3_1.fastq.gz<br>CTCF_IP_wt_rep3_2.fastq.gz<br>CTCF_IP_wt_rep4_1.fastq.gz<br>CTCF_IP_wt_rep4_2.fastq.gz<br>Rad21_input_BPTFko_rep1_1.fastq.gz<br>Rad21_input_BPTFko_rep1_2.fastq.gz<br>Rad21_input_BPTFko_rep2_1.fastq.gz<br>Rad21_input_BPTFko_rep2_2.fastq.gz<br>Rad21_input_BPTFko_rep3_1.fastq.gz<br>Rad21_input_BPTFko_rep3_2.fastq.gz<br>Rad21_input_wt_rep1_1.fastq.gz<br>Rad21_input_wt_rep1_2.fastq.gz<br>Rad21_input_wt_rep2_1.fastq.gz<br>Rad21_input_wt_rep2_2.fastq.gz<br>Rad21_input_wt_rep3_1.fastq.gz<br>Rad21_input_wt_rep3_2.fastq.gz<br>Rad21_IP_BPTFko_rep1_1.fastq.gz<br>Rad21_IP_BPTFko_rep1_2.fastq.gz<br>Rad21_IP_BPTFko_rep2_1.fastq.gz<br>Rad21_IP_BPTFko_rep2_2.fastq.gz<br>Rad21_IP_BPTFko_rep3_1.fastq.gz<br>Rad21_IP_BPTFko_rep3_2.fastq.gz<br>Rad21_IP_wt_rep1_1.fastq.gz<br>Rad21_IP_wt_rep1_2.fastq.gz<br>Rad21_IP_wt_rep2_1.fastq.gz<br>Rad21_IP_wt_rep2_2.fastq.gz<br>Rad21_IP_wt_rep3_1.fastq.gz<br>Rad21_IP_wt_rep3_2.fastq.gz<br>Wapl_IP_BPTFko_rep1_1.fastq.gz<br>Wapl_IP_BPTFko_rep1_2.fastq.gz<br>Wapl_IP_BPTFko_rep2_1.fastq.gz<br>Wapl_IP_BPTFko_rep2_2.fastq.gz<br>Wapl_IP_wt_rep1_1.fastq.gz<br>Wapl_IP_wt_rep1_2.fastq.gz<br>Wapl_IP_wt_rep2_1.fastq.gz

Wapl_IP_wt_rep2_2.fastq.gz
BPTF_IP_wt_rep1_1_read1.fastq.gz
BPTF_IP_wt_rep2_1_read1.fastq.gz
BPTF_IP_bptf_ko_rep1_1_read1.fastq.gz
BPTF_IP_bptf_ko_rep2_1_read1.fastq.gz
BPTF_input_wt_rep1_1_read1.fastq.gz
BPTF_input_wt_rep2_1_read1.fastq.gz
BPTF_input_BPTFko_rep1_1_read1.fastq.gz
BPTF_input_BPTFko_rep2_1_read1.fastq.gz
BPTF_IP_wt_rep1_1_read2.fastq.gz
BPTF_IP_wt_rep2_1_read2.fastq.gz
BPTF_IP_bptf_ko_rep1_1_read2.fastq.gz
BPTF_IP_bptf_ko_rep2_1_read2.fastq.gz
BPTF_input_wt_rep1_1_read2.fastq.gz
BPTF_input_wt_rep2_1_read2.fastq.gz
BPTF_input_BPTFko_rep1_1_read2.fastq.gz
BPTF_input_BPTFko_rep2_1_read2.fastq.gz

Wig files:
CTCF_input_BPTFko_rep1.wig.gz
CTCF_input_BPTFko_rep2.wig.gz
CTCF_input_BPTFko_rep3.wig.gz
CTCF_input_BPTFko_rep4.wig.gz
CTCF_input_wt_rep1.wig.gz
CTCF_input_wt_rep2.wig.gz
CTCF_input_wt_rep3.wig.gz
CTCF_input_wt_rep4.wig.gz
CTCF_IP_BPTFko_rep1.wig.gz
CTCF_IP_BPTFko_rep2.wig.gz
CTCF_IP_BPTFko_rep3.wig.gz
CTCF_IP_BPTFko_rep4.wig.gz
CTCF_IP_wt_rep1.wig.gz
CTCF_IP_wt_rep2.wig.gz
CTCF_IP_wt_rep3.wig.gz
CTCF_IP_wt_rep4.wig.gz
Rad21_input_BPTFko_rep1.wig.gz
Rad21_input_BPTFko_rep2.wig.gz
Rad21_input_BPTFko_rep3.wig.gz
Rad21_input_wt_rep1.wig.gz
Rad21_input_wt_rep2.wig.gz
Rad21_input_wt_rep3.wig.gz
Rad21_IP_BPTFko_rep1.wig.gz
Rad21_IP_BPTFko_rep2.wig.gz
Rad21_IP_BPTFko_rep3.wig.gz
Rad21_IP_wt_rep1.wig.gz
Rad21_IP_wt_rep2.wig.gz
Rad21_IP_wt_rep3.wig.gz
Wapl_IP_BPTFko_rep1.wig.gz
Wapl_IP_BPTFko_rep2.wig.gz
Wapl_IP_wt_rep1.wig.gz
Wapl_IP_wt_rep2.wig.gz
BPTF_IP_wt_rep1.wig.gz
BPTF_IP_wt_rep2.wig.gz
BPTF_IP_bptf_ko_rep1.wig.gz
BPTF_IP_bptf_ko_rep2.wig.gz
BPTF_input_wt_rep1.wig.gz
BPTF_input_wt_rep2.wig.gz
BPTF_input_bptf_ko_rep1.wig.gz
BPTF_input_bptf_ko_rep2.wig.gz

Genome browser session
(e.g. UCSC)

The following files can be uploaded to the UCSC genome browser by pasting the following URLs into “Paste URLs or data” in “add custom tracks”.
http://www.fmi.ch/groupdata/gschub/BPTF/CTCF_input_BPTFko_rep1.bw
http://www.fmi.ch/groupdata/gschub/BPTF/CTCF_input_BPTFko_rep2.bw
http://www.fmi.ch/groupdata/gschub/BPTF/CTCF_input_BPTFko_rep3.bw
http://www.fmi.ch/groupdata/gschub/BPTF/CTCF_input_BPTFko_rep4.bw
http://www.fmi.ch/groupdata/gschub/BPTF/CTCF_input_wt_rep1.bw
http://www.fmi.ch/groupdata/gschub/BPTF/CTCF_input_wt_rep2.bw
http://www.fmi.ch/groupdata/gschub/BPTF/CTCF_input_wt_rep3.bw
http://www.fmi.ch/groupdata/gschub/BPTF/CTCF_input_wt_rep4.bw
http://www.fmi.ch/groupdata/gschub/BPTF/CTCF_IP_BPTFko_rep1.bw
http://www.fmi.ch/groupdata/gschub/BPTF/CTCF_IP_BPTFko_rep2.bw
http://www.fmi.ch/groupdata/gschub/BPTF/CTCF_IP_BPTFko_rep3.bw
http://www.fmi.ch/groupdata/gschub/BPTF/CTCF_IP_BPTFko_rep4.bw
http://www.fmi.ch/groupdata/gschub/BPTF/CTCF_IP_wt_rep1.bw
http://www.fmi.ch/groupdata/gschub/BPTF/CTCF_IP_wt_rep2.bw
http://www.fmi.ch/groupdata/gschub/BPTF/CTCF_IP_wt_rep3.bw
http://www.fmi.ch/groupdata/gschub/BPTF/CTCF_IP_wt_rep4.bw
http://www.fmi.ch/groupdata/gschub/BPTF/Rad21_input_BPTFko_rep1.bw
http://www.fmi.ch/groupdata/gschub/BPTF/Rad21_input_BPTFko_rep2.bw
http://www.fmi.ch/groupdata/gschub/BPTF/Rad21_input_BPTFko_rep3.bw
http://www.fmi.ch/groupdata/gschub/BPTF/Rad21_input_wt_rep1.bw
http://www.fmi.ch/groupdata/gschub/BPTF/Rad21_input_wt_rep2.bw
http://www.fmi.ch/groupdata/gschub/BPTF/Rad21_input_wt_rep3.bw
http://www.fmi.ch/groupdata/gschub/BPTF/Rad21_IP_BPTFko_rep1.bw
http://www.fmi.ch/groupdata/gschub/BPTF/Rad21_IP_BPTFko_rep2.bw
http://www.fmi.ch/groupdata/gschub/BPTF/Rad21_IP_BPTFko_rep3.bw
http://www.fmi.ch/groupdata/gschub/BPTF/Rad21_IP_wt_rep1.bw
http://www.fmi.ch/groupdata/gschub/BPTF/Rad21_IP_wt_rep2.bw
http://www.fmi.ch/groupdata/gschub/BPTF/Rad21_IP_wt_rep3.bw
http://www.fmi.ch/groupdata/gschub/BPTF/Wapl_IP_BPTFko_rep1.bw
http://www.fmi.ch/groupdata/gschub/BPTF/Wapl_IP_BPTFko_rep2.bw
http://www.fmi.ch/groupdata/gschub/BPTF/Wapl_IP_wt_rep1.bw
http://www.fmi.ch/groupdata/gschub/BPTF/Wapl_IP_wt_rep2.bw
http://www.fmi.ch/groupdata/gschub/BPTF/BPTF_IP_wt_rep1.bw
http://www.fmi.ch/groupdata/gschub/BPTF/BPTF_IP_wt_rep2.bw
http://www.fmi.ch/groupdata/gschub/BPTF/BPTF_IP_bptf_ko_rep1.bw
http://www.fmi.ch/groupdata/gschub/BPTF/BPTF_IP_bptf_ko_rep2.bw
http://www.fmi.ch/groupdata/gschub/BPTF/BPTF_input_wt_rep1.bw
http://www.fmi.ch/groupdata/gschub/BPTF/BPTF_input_wt_rep2.bw
http://www.fmi.ch/groupdata/gschub/BPTF/BPTF_input_bptf_ko_rep1.bw
http://www.fmi.ch/groupdata/gschub/BPTF/BPTF_input_bptf_ko_rep2.bw

Methodology

Replicates

From two to four biological replicates were performed.

Sequencing depth

Sample_Name	Total_reads	Mapped_reads
CTCF_IP_wt_rep1	35192624	32224764
CTCF_IP_wt_rep2	39928904	36950512
CTCF_IP_BPTFko_rep1	39502118	36482367
CTCF_IP_BPTFko_rep2	38256394	35329755
CTCF_input_wt_rep1	34740264	32609634
CTCF_input_wt_rep2	19397729	18238325
CTCF_input_BPTFko_rep1	35408279	33264992
CTCF_input_BPTFko_rep2	20609623	19337074
CTCF_IP_wt_rep3	41275856	38403430
CTCF_IP_wt_rep4	37015816	34646028
CTCF_IP_BPTFko_rep3	40169605	36915807
CTCF_IP_BPTFko_rep4	38298894	35359970
CTCF_input_wt_rep3	29157171	27482691
CTCF_input_wt_rep4	33984356	32132314
CTCF_input_BPTFko_rep3	26673398	25246762
CTCF_input_BPTFko_rep4	32079806	30329742
Rad21_IP_wt_rep1	42831513	40228789
Rad21_IP_wt_rep2	39772331	37385655
Rad21_IP_wt_rep3	42121353	39523083
Rad21_IP_BPTFko_rep1	36729473	34371224
Rad21_IP_BPTFko_rep2	39770249	37327224
Rad21_IP_BPTFko_rep3	40226022	37740551
Rad21_input_wt_rep1	32985598	31124741
Rad21_input_wt_rep2	32082857	30210595
Rad21_input_wt_rep3	34328803	32405497
Rad21_input_BPTFko_rep1	34306857	32357374

Rad21_input_BPTFko_rep2 37288033 35147322
Rad21_input_BPTFko_rep3 35198271 33103882
Wapl_IP_wt_rep1 59577081 56016345
Wapl_IP_wt_rep2 35996719 33389039
Wapl_IP_BPTFko_rep1 51526126 48419132
Wapl_IP_BPTFko_rep2 42275419 39736154
Bptf_IP_wt_rep1 128961468 92409710
Bptf_IP_wt_rep2 113898972 81045910
Bptf_IP_BPTFko_rep1 155104492 111819052
Bptf_IP_BPTFko_rep2 120492214 90475200
Bptf_input_wt_rep1 82182150 69150740
Bptf_input_wt_rep2 88491534 73008342
Bptf_input_BPTFko_rep1 76505840 63308318
Bptf_input_BPTFko_rep2 74983422 63334034

Antibodies

CTCF-C-20X-Santa Cruz, Rad21-ab992-Abcam, Wapl-16370-1-AP-Proteintech, Bptf- ABE24-Millipore.

Peak calling parameters

Specify the command line program and parameters used for read mapping and peak calling, including the ChIP, control and index files used.

Data quality

ChIP-seq sample quality was assessed using the following criteria:
- technical quality (sufficient sequencing depth and unique-hit mapping rates)
- reproducibility between biological replicate samples

Software

Analysis was performed using R 4.3.0 and R/Bioconductor packages: QuasR 1.40.1.
