# Supplementary figures and images for: Systematic assessment of ISWI subunits shows that NURF creates local accessibility for CTCF
Source: Nat Genet. 2024 May 30;56(6):1203–12. doi: 10.1038/s41588-024-01767-x (PMC11176080; doi:10.1038/s41588-024-01767-x)

# Unprocessed Western blot images for Fig 1b

ACF1

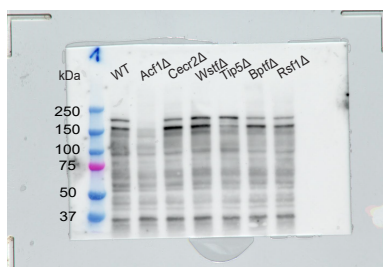

CECR2

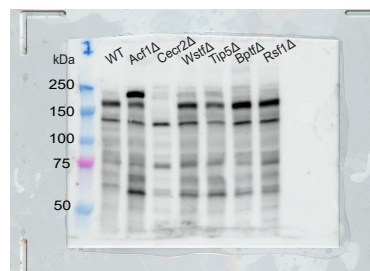

WSTF

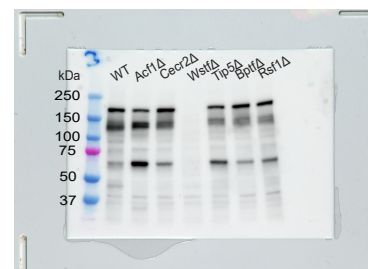

TIP5

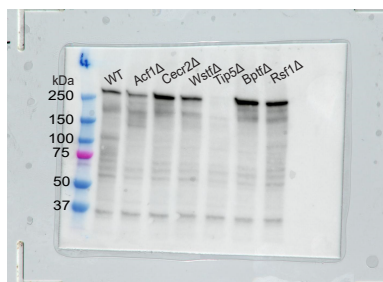

BPTF

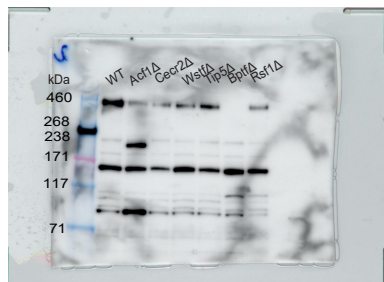

RSF1

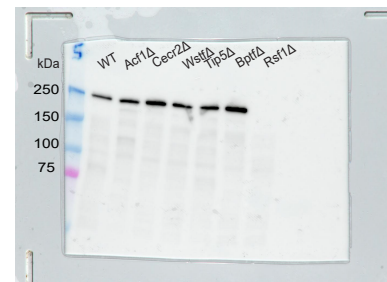

SNF2H

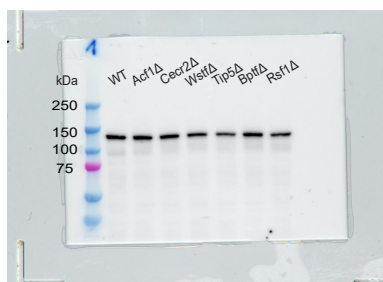

LAMINB

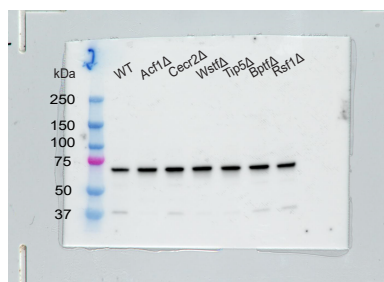

CTCF (on TIP5 blot)

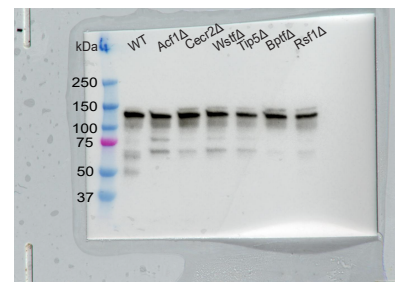

Supplement: Supplementary file 5 — Unprocessed western blots. [file 41588_2024_1767_MOESM5_ESM.pdf]
