# Supplementary material for: Systematic assessment of ISWI subunits shows that NURF creates local accessibility for CTCF
Source: Nat Genet. 2024 May 30;56(6):1203–12. doi: 10.1038/s41588-024-01767-x (PMC11176080; doi:10.1038/s41588-024-01767-x)

Unprocessed Western blot images for Extended Data Fig2a

ACF1

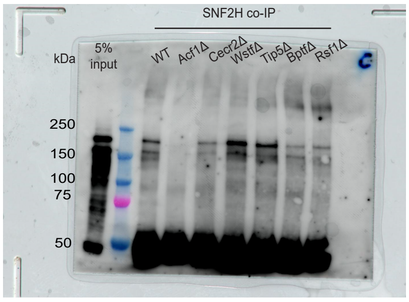

CECR2

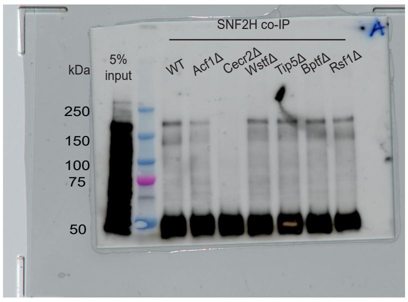

WSTF

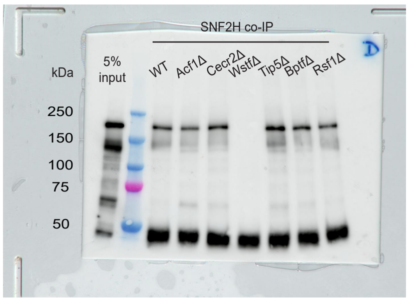

TIP5

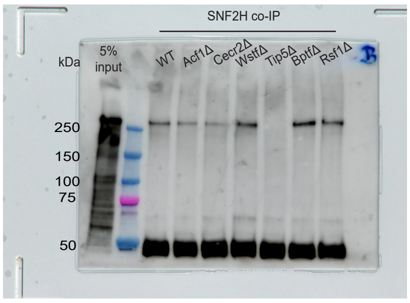

BPTF

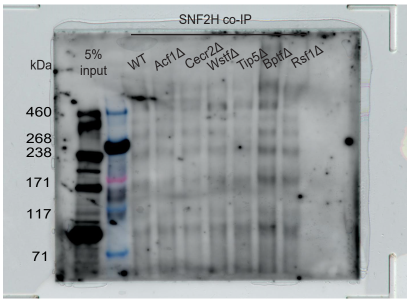

RSF1

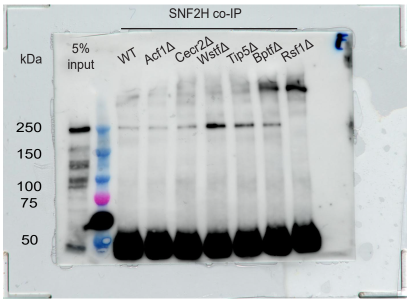

SNF2H (on WSTF blot)

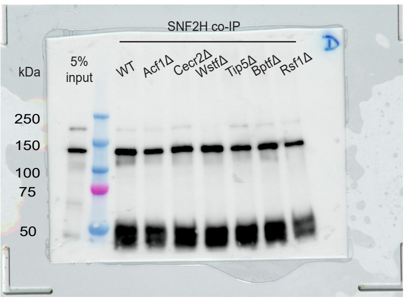

Supplement: Supplementary file 7 — Unprocessed western blots. [file 41588_2024_1767_MOESM7_ESM.pdf]
