# Supplementary material for: Systematic assessment of ISWI subunits shows that NURF creates local accessibility for CTCF
Source: Nat Genet. 2024 May 30;56(6):1203–12. doi: 10.1038/s41588-024-01767-x (PMC11176080; doi:10.1038/s41588-024-01767-x)

Unprocessed Western blot images for Extended Data Fig10f

RAD21 (blot on the left)

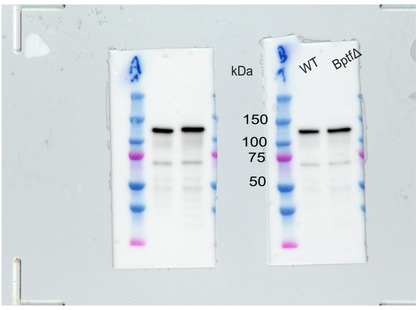

LAMINB (blot on the left)

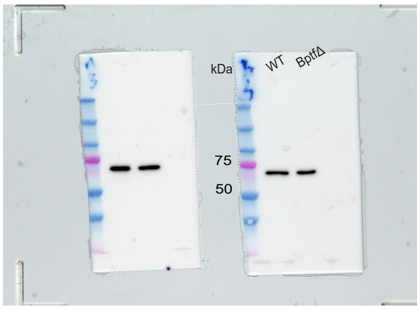

Supplement: Supplementary file 8 — Unprocessed western blots. [file 41588_2024_1767_MOESM8_ESM.pdf]
